# Distributional Learning of Appearance

**DOI:** 10.1371/journal.pone.0058074

**Published:** 2013-02-27

**Authors:** Lewis D. Griffin, M. Husni Wahab, Andrew J. Newell

**Affiliations:** 1 Computer Science, University College London, London, United Kingdom; 2 Complex, University College London, London, United Kingdom; The University of Plymouth, United Kingdom

## Abstract

Opportunities for associationist learning of word meaning, where a word is heard or read contemperaneously with information being available on its meaning, are considered too infrequent to account for the rate of language acquisition in children. It has been suggested that additional learning could occur in a distributional mode, where information is gleaned from the distributional statistics (word co-occurrence etc.) of natural language. Such statistics are relevant to meaning because of the Distributional Principle that ‘words of similar meaning tend to occur in similar contexts’. Computational systems, such as Latent Semantic Analysis, have substantiated the viability of distributional learning of word meaning, by showing that semantic similarities between words can be accurately estimated from analysis of the distributional statistics of a natural language corpus. We consider whether appearance similarities can also be learnt in a distributional mode. As grounds for such a mode we advance the Appearance Hypothesis that ‘words with referents of similar appearance tend to occur in similar contexts’. We assess the viability of such learning by looking at the performance of a computer system that interpolates, on the basis of distributional and appearance similarity, from words that it has been explicitly taught the appearance of, in order to identify and name objects that it has not been taught about. Our experiment tests with a set of 660 simple concrete noun words. Appearance information on words is modelled using sets of images of examples of the word. Distributional similarity is computed from a standard natural language corpus. Our computation results support the viability of distributional learning of appearance.

## Introduction

**Figure 1:**
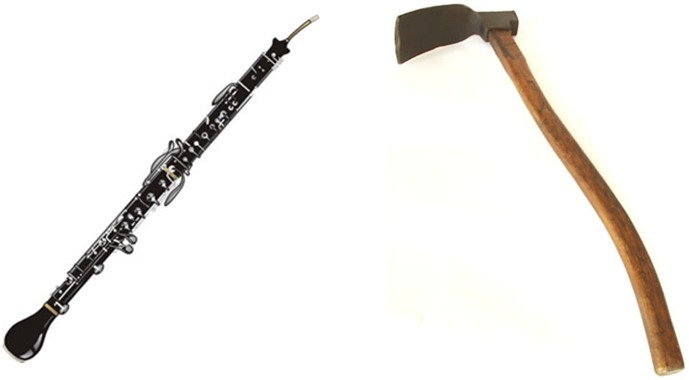
Objects from unfamiliar categories. On the left a *cor anglais* (a woodwind instrument), on the right an *adze* (a woodworking tool).

We start with an informal motivation. For many viewers the two objects shown in Figure 1 are unfamiliar; but if asked which is an *adze* most will get it right. This seems to be an act of visual identification without it being one of visual recognition. Asked how they knew, a typical account is: they had heard the word before, were not sure what it was, but from the context in which it was said they thought that it was some kind of tool, perhaps used in heavy work; so they choose the object that looked more like the tools used in heavy work which they were familiar with such as axes or mauls. The heuristic which seems to underwrite this process is:

Appearance Hypothesis (AH): *words that occur in similar contexts tend to have referents with similar appearance*

We hypothesise that children make use of the AH when they are learning the meaning of words. Consider a child who does not know what a *pear* looks like. When she starts to hear the word used she can track the company it keeps and deduce, by application of the AH, some words whose meaning she does know that should look similar; and thus develop a an approximate idea of its appearance. When the child sees something that looks more like this idea than it looks like any category that she does know she can venture that it is a *pear*. She can then start to build up an understanding of the appearance of *pear* based on direct experience rather than generalization, perhaps after seeking confirmation for the guess by trying out the word *pear* at a suitable juncture. We will return repeatedly to this example.

Of course, whether such a mechanism *is* used in human language acquisition can only truly be tested through experiments with children, but it could be ruled out if it was shown that the AH was ineffective either because it was false or very weak. In this paper we make a computational assessment of the AH to see if it can be ruled out. The computational assessment will require us to combine methods most commonly encountered within cognitive science and within computer science. From cognitive science we will use methods that measure the similarity of the contexts within which words are found based on a representative corpus of natural language. From computer science we will use methods that compute similarities between images, and similarities between categories of objects based on images of them.

Before the computational section, in the remainder of the introduction, we give a more formal motivation and discussion of previous related research. We start with a discussion of word meaning as a component of semantic knowledge. We consider the puzzle of the rapid rate of acquisition of word meanings (i.e. language) exhibited by children and describe two modes for this acquisition: associationist where an instance is clearly labelled by language (‘this is a lion’ or ‘a chapel is a small house of worship usually associated with a main church’) and distributional where meaning is extracted from the statistics of words heard in passing. We then consider the narrower problem of learning the visual appearance part of word meaning, and how this can take place in the associationist and distributional modes of language acquisition. We review methods used in machine vision to learn appearances in case it has any special lessons for the process in humans.

### Semantic Knowledge, Language & Meaning

Human cognition organizes the world into categories (e.g. cats, bridges, theorems). Knowledge relating to categories is called semantic. Elements of semantic knowledge can be of internal or external type. Those of internal type are specific to individual categories and come in a variety of characters: perceptual (e.g. dogs look like *S*), motoric (e.g. buttons move like *M*) or amodal (e.g. London is the capital city of the UK). Those of external type interrelate multiple categories (e.g. a finger is part of a hand, a cap is a kind of clothing, zebras look like horses, a scallop is akin to a mussel) [1].

Via the relation of *meaning,* language connects to the categories that human cognition imposes on the world. The nature of the relationship is subtle and has been discussed in Philosophy and Psychology. Some points of argument established within Philosophy are: (i) meaning within a brain and within a language-using community may need to be distinguished [2]; (ii) there may be a distinction between the referents of a word and the connection of the word to those referents [3]; (iii) the connection may be via a descriptive criteria [4] or through a causal relationship [5]. Within Psychology the emphasis of enquiry has been on: meaning for individuals; the implementation of meaning through the relations that a word holds with other mental items; and the acquisition of meanings. Regarding the implementation of meanings within brains, two possibilities have been suggested: through relations involving only words, and through relations between words and sense or motor data. It is generally agreed that it is untenable for the meaning of all words to arise from word relations alone, as then a connection to the external world would either be lacking [6] or at least of the wrong sort [7]. Regarding acquisition of meanings, more commonly called language acquisition, a crucial datum that needs to be explained is the rate: modern adult humans have semantic knowledge of between 30,000 [8] and 70,000 [9] categories, hence acquired at an average rate of 10 per day during childhood years.

### Associationist Language Acquisition

Accounting for the rate of language acquisition is taken to be an instance of Plato’s problem, more generally referring to apparent gaps between the richness of knowledge and the paucity of opportunities for learning afforded by the environment [10]. The task for Psychology is to identify modes of learning which are jointly adequate to bridge the gap.

The most obvious mode for learning the meaning of a word is *direct associationist* i.e. perceptual experience of an example along with a label (e.g. ‘look a tiger’). Unambiguous labellings are no doubt desirable but not strictly necessary as children are known to be expert at inferring what a speaker is referring to [11]; using heuristics such as excluding as candidates anything the child already knows the name of [12]. Moreover, the label need not always be co-present with the referent on all occasions. Algorithms for semi-supervised learning that cover such cases have been developed in Psychology [13] and in Computer Science [14]. The general idea is that a learner can learn an approximate meaning for a word based on a small number of instances of associationist learning, can then use that approximate meaning to identify unlabelled instances of the word, and can then use these further instances to refine the learnt meaning, and so on. So long as the labels are not too often applied erroneously, the learnt meaning will incrementally improve.

*Indirect associationist* acquisition of meaning occurs when only a proxy for the referent of the word is present. The proxy can be a model, a photograph, an illustration etc. or a linguistic description or definition. This is the mode used when one learns from the statement ‘a petard is a small bomb used to blow up gates and walls when breaching fortifications’. As already noted, there would be a ‘grounding’ problem if all meanings were learnt like this [6], but this is not a problem in practice.

Even with these extensions and variants the associationist mode seems insufficient to bridge the gap; and no variety of it accounts for the *adze* example we gave earlier, where a meaning for a word (rough and ready by all means, but good enough to allow visual identification in the right circumstances) seems to have been acquired simply through hearing the word used without it ever being present, described or defined.

### Distributional Language Acquisition

It has been suggested that, in addition to the direct and indirect associationist modes by which language can facilitate the acquisition of word meanings, there is a further *distributional* mode. In such a distributional mode, meaning is not delivered in discrete morsels (e.g. ‘look, a tiger!’ or ‘a petard is a small bomb used…’), but instead in statistical patterns, weakly and diffusely present, across bodies of natural language. This is the mode we were indicating when we described in the opening paragraph to this paper how a subject might explain their understanding of a word such as *adze* – “they had heard the word before, were not sure what it was, but from the context in which it was said they thought that it was some kind of tool, perhaps used in heavy work”.

The possibility of such a distributional mode of learning rests upon the following, originating in the works of Harris, Firth and Weaver [15]–[17]:

Distributional Principle (DP): *words that occur in similar contexts tend to have similar meanings.*

Explicit tests of the DP, first by Rubenstein & Goodenough [18], later by Landauer & Dumais [19] and most recently by Rohde et al. [20], have found good support for it. These studies vary in how ‘context’ and ‘similarity of contexts’ is defined. Contexts may be defined by windows of fixed length (e.g. ±4 words) around an instance of a word, with flat or tapered weighting; or they may be defined more syntactically, for example within the containing sentence or paragaraph. Within a context the occurrence of all words may be tracked, or stop words such as ‘the’ may be ignored. Occurrence counts may be transformed in numerous ways e.g. by log-transformation. Vectors of possibly-transformed occurrence counts can be projected into lower-dimensional spaces. Finally, context feature vectors can be compared using Euclidean distances, inner products or correlation.

Of the various methods of defining contexts and their similarity, the Latent Semantic Analysis (LSA) method introduced by Landauer & Dumais deserves special comment. LSA is remarkable for two reasons. First that it introduced the use of a dimensionality reduction step in the processing of context distribution descriptors. Second that it has been a very impactful method, and an important stimulus in the rise of the technologically significant field of computational semantics [21]. A recent comparison of methods of distributional similarity shows that dimension reduction is a useful component but not of decisive importance [20]. The comparative study produced overall figure-of-merit scores based on a battery of 17 lexical-semantic tasks. Across the 15 methods compared, scores ranged from 26.4 for the HAL-400 model derived from the work of Burgess & Lund [22], up to 73.4 for the study authors’ method COAL-SVD-800 which uses the best of everything including dimensionality reduction. A pure LSA method scored 61.6, while the authors’ method without dimensionality reduction (COALS-14K) scored 69.2.

Although suggestive, the power of methods such as COALS and LSA that infer semantic similarities from distributional ones, does not mean that distributional learning is used in human language acquisition. Experimental evidence that pertains to this issue is scarce. The most relevant are results which showed that the semantic similarity of words can be effected by manipulating the contexts in which the words appear [23]. Although supportive of distributional learning in humans, these results are for adult subjects and so the relevance for the main phase of language acquisition can be doubted.

### Human Learning of Appearance

The previous two sections were concerned with mechanisms for learning word meaning in general. We now narrow the focus to a particular aspect of meaning – visual appearance – and consider how that may be learnt.

Learning visual appearances in associationist mode is complex but it is not contentious that it does occur. In direct associationist mode, when an instance is present physically or pictorially, invariant encodings of sense data may be compiled into semantic knowledge and linked to the heard or read label. In indirect associationist mode, when the referent is present only linguistically (e.g. ‘a griffin is a lion with eagle’s wings’), there are several plausible possibilities: the information may be stored linguistically; an invariant sense data encoding may be directly constructed; or sense data may be synthesised through an imaginative process, and an invariant encoding constructed from it.

The concern in this paper is whether the appearance parts of word meanings can also be learnt in a distributional mode. We believe that they can, at least partially. What makes it possible is the principle stated at the beginning of the paper and now restated.

Appearance Hypothesis (AH): *words that occur in similar contexts tend to have referents with similar appearance*

The AH provides the basis for a mechanism to learn appearances in a distributional mode. Our example scenario is a child who does not yet know the appearance of *pear*. The child could attend to the words surrounding *pear* in speech and text (i.e. the contexts); could summarize the (distributional) statistics of these contexts; and could then compare these statistics to those of words which she did know the appearance of. She might (for example) realize that the distributional statistics of *pear* were similar to those of *apple*; and dis-similar to those of *train*. Then, when some unfamiliar object presented itself, which was sufficiently similar in appearance to *apple* and/or sufficiently dis-similar to *train,* she could apply the AH and guess that the object was a *pear*, and then either assume that the guess was correct and treat the incident like a regular opportunity for associationist learning, or more cautiously try saying the word looking for confirmation.

**Figure 2:**
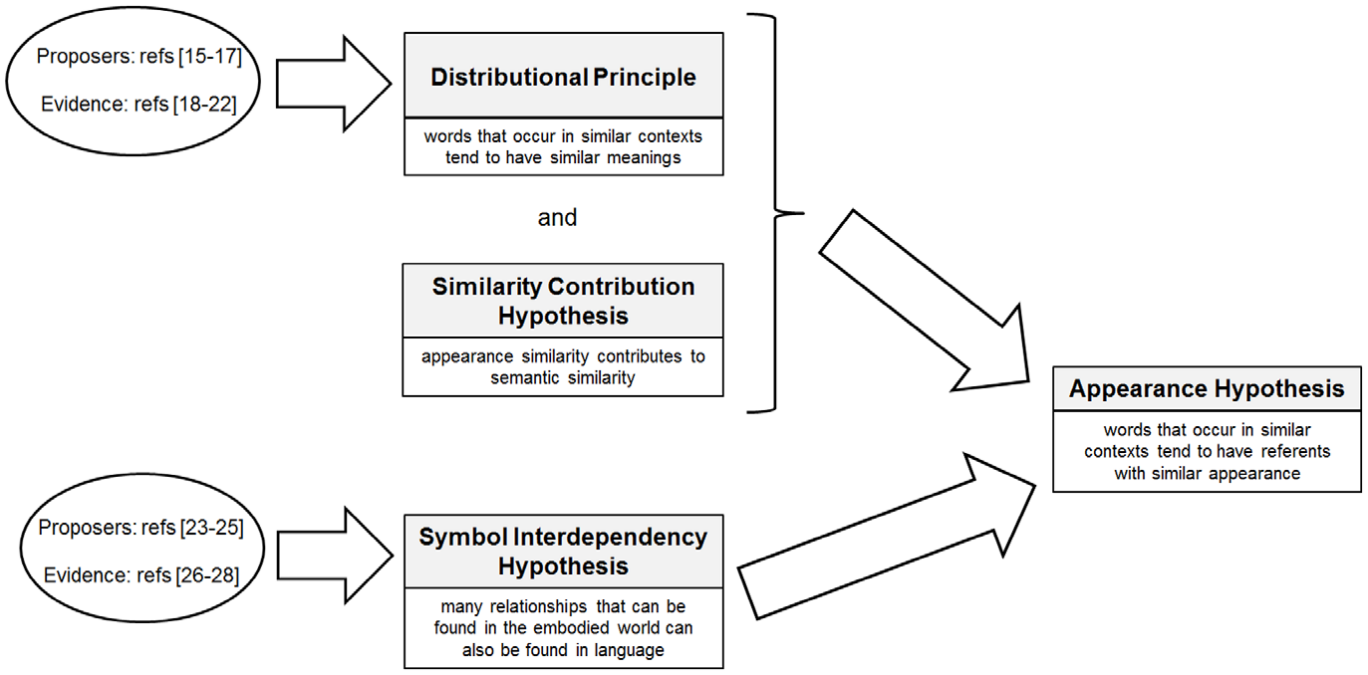
Arguments leading to the Appearance Hypothesis.

Our aim in this paper is to state and to test the AH, but we also consider whether there are already established grounds to believe it. We illustrate two possible arguments in figure 2. The first argument (upper route of figure 2) builds on the DP and has been expressed by Landauer & Dumais as follows:

“Because, purely at the word level, *rabbit* has been indirectly preestablished to be something like *dog, animal, object, furry, cute, fast, ears,* etc., it is much less mysterious that a few contiguous pairings of the word with scenes including the thing itself can teach the proper correspondences. LSA could easily learn that the words *rabbit* and *hare* go with pictures containing rabbits and not to ones without, and so forth.” [19]

The logic being used here is to combine the DP with the following additional (implicit) hypothesis:

Similarity Contribution Hypothesis (SCH): *appearance similarity contributes to semantic similarity*

to reach the conclusion AH. We note that (i) SCH is a substantive claim – if visual appearance was assessed by retail barcode (a reasonable strategy for a warehouse robot for example) then it would not be true; and (ii) although SCH is intuitively reasonable, hard evidence for it is lacking.

The second argument (lower route of figure 2) proceeds from different premises. It is founded on the:

Symbol Inter-dependency Hypothesis (SIH): *language is structured in such a way that many relationships that can also be found in the embodied world are structured in language. Language thereby provides a shortcut to the embodied relations in the world* [24], [25].

The SIH can be traced back to C.S. Pierce [26] but has been more recently elaborated by Louwerse [24], [25]. Evidence supportive of the SIH includes: the correlation between the length of words and the rarity of their referents [27]; word order reflecting spatial layout [28]; and that the co-occurrence statistics of adjectives are predictive of their modality [29].

We see three advantages to the argument for the AH based on the SIH (lower route) over that based on the DP (upper route): (i) no unproven supplementary premise is needed, (ii) the possibly troublesome concept of semantic similarity is not used, and (iii) we avoid the disconcerting step of inferring a weak correlation between *D* and *A* from weak correlations between *D* and *S*, and *S* and *A*. On the other hand, the argument based on DP avoids the SIH, and the SIH while fascinating is broad, possibly vague and definitely contentious. Regardless of which prior grounds for the AH are preferred, for our computational experiments we simply test the AH against the evidence.

### Machine Learning of Appearance

The gap between experience and knowledge does not seem as challenging for Machine Vision as it does for Human Vision. The difference is the possibility of tireless associationist learning. For example, supervised learning algorithms [30] that can learn the appearance of an object category from at least 10, ideally 10^3^, labelled examples have been developed [31]. Implemented for the adult repertoire of categories such an approach requires around 10^7^ labelled training images. With the advent of the internet, assembling such a database is now possible.

Reliably labelled databases with 10^7^ images are constructable by manual means. Databases larger than this can be assembled using automated methods but the labels will inevitably be incomplete, erroneous and ambiguous. Methods that can learn from such poorly labelled data are being developed. For example, as learning proceeds the training images can be refined by progressively removing poorly labelled images [32], and by progressively localizing objects within the images [33]. There are also approaches that train many categories in parallel, and are therefore able to deal with images with multiple labels only one of which is correct [34], or to pick the best label out of a range of alternatives [35]. There are even approaches that combine all of the above to deal with images with multiple objects and multiple labels [36]. Additionally, methods of semi-supervised learning, as described earlier, can make use of completely unlabelled data, so long as there is some labelled data to initialize the process [37].

While the most dramatic advances in Computer Vision are currently coming from scaling up the associationist mode, there are other methods being developed that do not fit into that mould. They all aim at some form of cross-category generalization but are diverse in nature and vary in their locus of application. They include:

- optimizing the low-level features used as the basis of identification across a set of categories rather than separately for each category [38];
- optimizing a decision tree for categorization [39], [40];
- identifying an object on the basis of its pattern of similarities to 2000+ fixed categories [41];
- identifying a category on the basis of detection of attributes (e.g. striped, lives in water), the detection of which is trained across categories [42], [43];
- and effectively increasing training data by treating an image labelled with one category as also being a weakly-labelled instance of a semantically-related category [44].

## Methods

**Figure 3:**
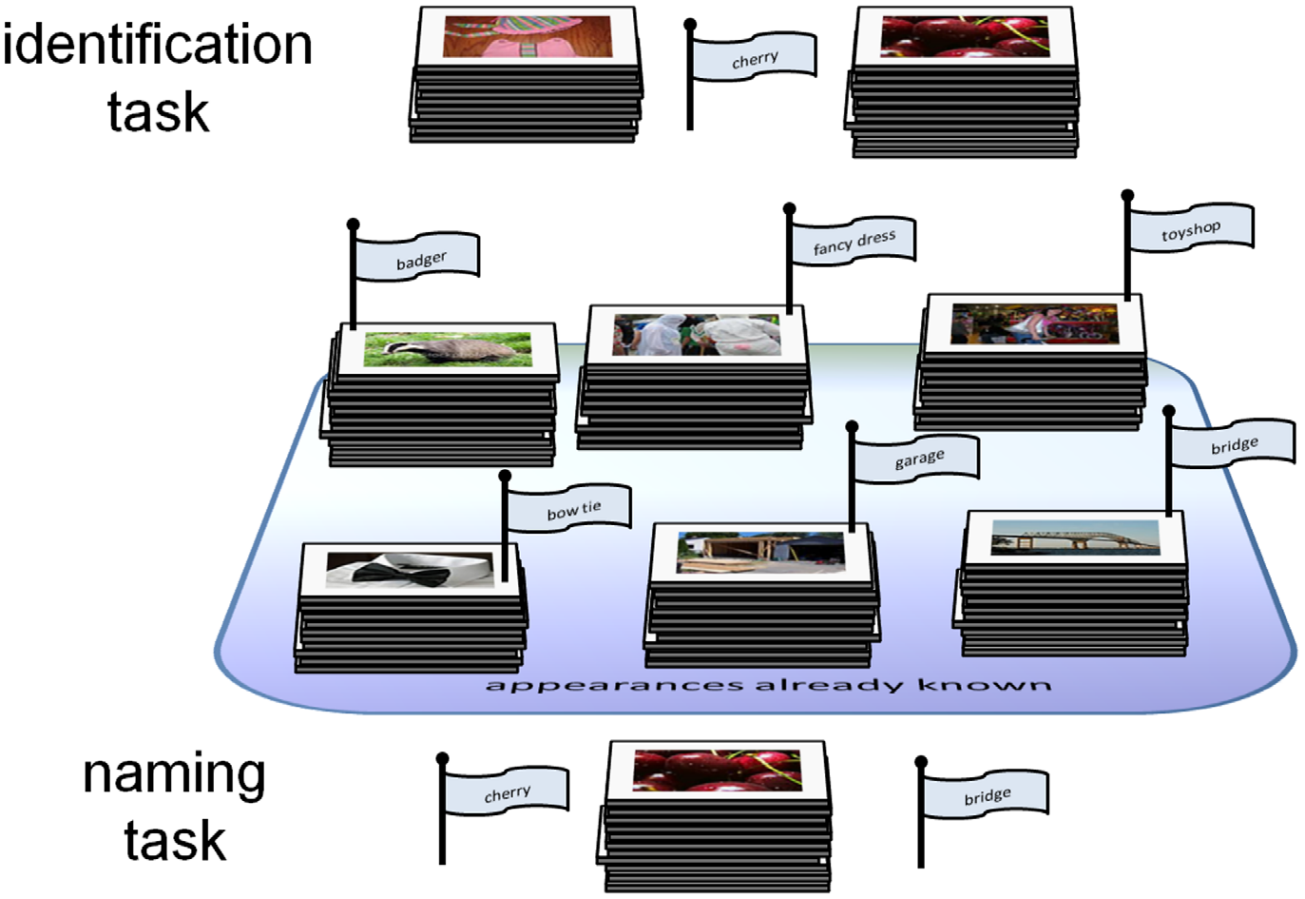
The identification and naming tasks. In our experiments, the appearance of a word is modelled using a set of images of examples of that word, which in the figure we illustrate as a pile of images. The identification task is to determine which of two unlabelled novel appearances should be paired with a word (in this case *cherry)*. The correct answer is on the right. The naming task is to determine which of two labels (in this case *cherry* and *bridge*) should be paired with an appearance. The correct answer is *cherry*. In both types of task the system is provided with knowledge of the appearance of a set of words (in the figure six) disjoint from those involved in the task.

We wish to assess whether the tendency expressed in the Appearance Hypothesis (AH) is sufficiently strong to be the basis of distributional learning of appearance. We do this by constructing a computational model learning system and testing whether it can acquire knowledge where it has had no associationist opportunity to do so. The AH is supported if the system performs significantly better than chance at an identification task and a naming task, both illustrated in figure 3.

For either type of task. the system is prepared for testing by simulating periods of (i) associationist learning of various appearances, not including for two particular test words (e.g. *cherry* and *bridge*); and (ii) exposure to natural language from which it can extract distributional statistics. For the identification task, we then present the system with unlabelled appearance data for *cherry* and for *bridge* and have it make its best guess at identifying which of the appearances is of *cherry*. For the naming task we present it with a single unlabelled appearance (say *cherry*) and have it make its best guess as to whether the appearance is that of *cherry* or *bridge*.

Our model learning system has three data and three algorithmic components. The data components are:

- a corpus of natural language for computation of distributional similarities,
- a set of words whose appearances have to be learnt, and
- appearance data in the form of images for the testing words

The algorithmic components are:

- an image-based measure of appearance similarity,
- a measure of distributional similarity, and
- algorithms for the identification and naming tasks based on appearance and distributional similarities.

### Data

As the basis for computing distributional similarities, we used the British National Corpus (BNC) [45] which is made up of written texts and transcribed speech. The text has been pre-processed to remove punctuation, parentheses and unclear utterances; and the words of the text have been converted to standardized word tokens with consistent conjugation, pluralisation etc. (e.g. ‘mouse’ replaces ‘mice’). This yields 4.2×10^5^ distinct word tokens spread over 9.6×10^7^ words. The 1^st^, 10^th^, 100^th^, 1000^th^, 10,000^th^ and 100,000^th^ most common word tokens are ‘the’, ‘he’, ‘between’, ‘sorry’, ‘tenor’ and ‘uniimog’; and they occur 6.0×10^6^, 1.2×10^6^, 9.1×10^4^, 1.1×10^3^, 420 and 6 times respectively. The words we use in the experiment (*W_660_*) occur in the corpus with varying frequency. The rarest is ‘jack-in-the-box’ which occurs 12 times, the most common is ‘people’ which occurs 1.2×10^5^ times. The median frequency is 1436.

For words which our system will learn the appearances of we used a diverse set (denoted *W_660_*) of 660 categories taken from a children’s vocabulary picture book [46]. Examples are: *starfish*, *bus*, *airport*, *hole* and *house*. The 660 were chosen from the 1000 in the source reference by taking only nouns, with single word names, and with entries in WordNet [9]. For use in supplementary computations, the words were grouped by the authors into 21 categories, for example *ANIMAL* and *TRANSPORT*. We also defined two nested subsets of the main set of categories: 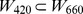 are the categories that have image collections in ImageNet [47]; and 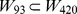 are the categories for which ImageNet also provides encodings of the images in terms of the widely-used SIFT local image descriptor [48].

For each word in *W_660_* we collected 50 images using the ‘Google Images’ web search tool. For words in *W_420_* we also collected the 50 images from the ImageNet database [47]. Images were retrieved from Google Images using searches with options enabled to return only full-colour, jpeg-encoded, photo images. 1% of images were found to be exact or near-duplicates. After removing these, the first 50 images return by the search were used. The thumbnail versions of the images, made by Google, were used rather than the originals. Their mean size was 108×123 pixels. For ImageNet, the first 50 colour images in each category were used, resized to thumbnails.

### Appearance Similarity

There are many ideas about how the appearance of a category is represented neutrally: for example: feature lists, prototypes, or unanalyzable neural nets; each of which can be concerned with object-centred or view-based descriptions of individual objects. In our experiment we model the appearance aspect of word meaning using sets of images showing different example referents of the word. This is similar to a multiple prototype model of categories in cognitive science [49], and, in the sphere of machine learning, to a nearest neighbour approach [50] where examples of the data are used as the model of the population. Because we use an image rather than a 3-D geometrical model for each prototype, our representation is of the view-based type, rather than the object-centred [51].

We use a set of 50 images to model the appearance of each category. The images within a set vary in viewpoint, lighting and surrounding context as well as showing different instances of the category. Jointly the images in a set characterize the distribution of perceptual impressions that referents of a word may give rise to.

We distinguish between appearance similarity, which relates two words, and image similarity, which relates two images. We define appearance similarity in terms of image similarity. The similarity between two appearances is the mean similarity between each image in an appearance set to the most similar image in the other appearance set.

**Figure 4:**
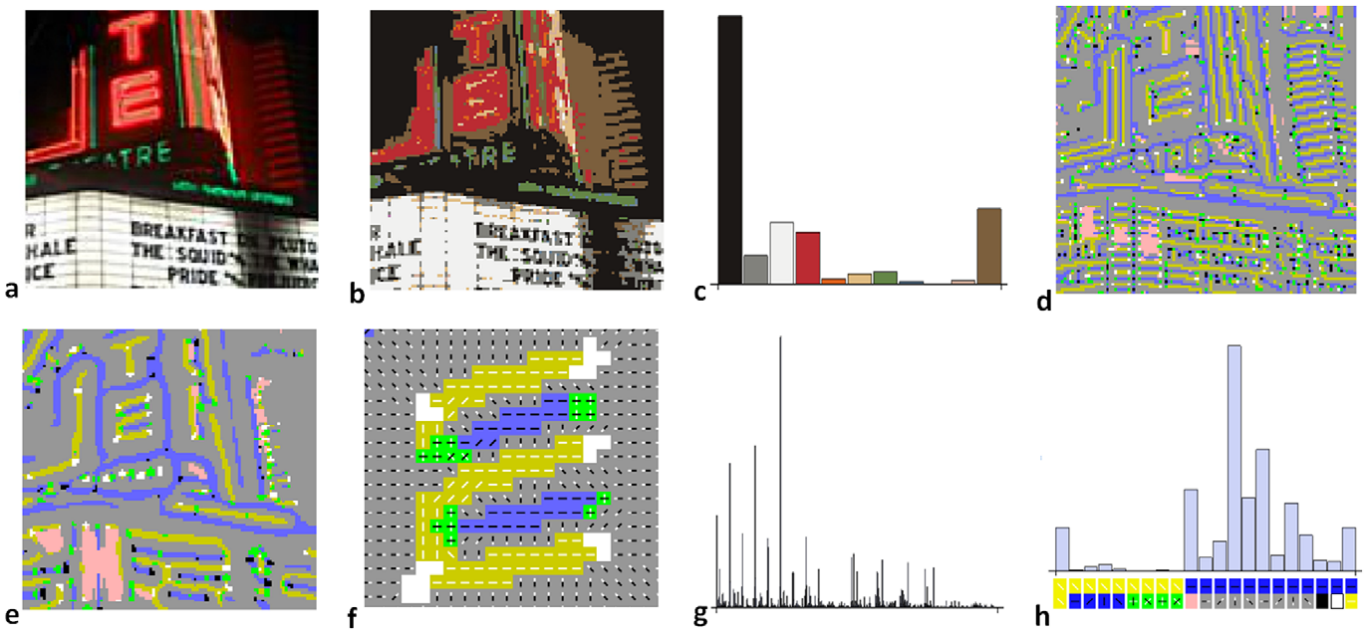
Image encodings used. (**a**) an example image for the category *cinema*, (**b**) each pixel is classified as one of the eleven Basic Colours, (**c**) the histogram of these is the color-based image encoding; (**d,e**) each pixel is classified as one of the 23 oriented Basic Image Features (oBIFs) at a fine and a coarse scale (**f**) a detail from e (slightly north-west of centre) showing the orientations of the oBIFs, (**g**) the histogram of ordered pairs of fine and coarse scale oBIFs is the texton-based image encoding, (**h**) a zoomed detail from g.

The measures of image similarity that we use are based on histogram type encodings of the images (see figure 4). Histogram encodings give detailed counts of the micro-elements that appear within an image, but give no information on how these elements are arranged. We use colour histograms and texton histograms. Colour histograms represent the distribution of colours present within an image e.g. 7% black, 1% red, 12% brown, etc. Texton histograms represent the distribution of local structural elements present within the image e.g. 3.1% horizontal dark line segments, 0.8% light blobs, 0.3% T-junctions etc. We also present results when both types of encoding are used together, with the expectation that this will give performance greater than each individually.

Our use of histogram encodings for images parallels the use of bag-of-word type encodings for text analysis. In both cases, information about the counts of different elements present is retained, while information on their arrangement is discarded. In both cases, even though the discarded arrangement information is expected to be extremely rich, performance which is surprisingly sensitive to semantic content has still been obtained [21], [52]–[54]. It is not difficult to produce examples for images [55] and text [56] where the discarded information is crucial; and it is widely believed that a new generation of encodings, which are sensitive to texton arrangement [57] and word order [58], will eventually lead to improved performance in systems that automatically determine the semantic content of images and documents. At present though the incremental performance for these more advanced systems is relatively modest, and at the expense of considerable increase in algorithm complexity and computational cost.

Histogram encodings use the counts recorded in a system of bins that partition the relevant feature space. In text analysis the space is words, and there is consensus that good bins are sets of words that have the same stemmed form. In image analysis, comparison of binning systems is still an active area of research [59]. In this work we use bin systems that we have developed elsewhere. For colour-based image encoding we used a system of 11 bins corresponding to the Basic Colours (black, grey, white, red, orange, yellow, green, blue, purple, pink, brown) [60]. Each bin is a connected region of the RGB cube; they are disjoint and their union is the full cube. We have previously shown that this is a simple and effective colour encoding with grip on semantic content [61]. For texton-based image encoding we use a system of 529 bins which partition the space of possible local image patches. We have previously shown that encoding images using a histogram of these textons gives state-of-the-art performance on match-to-sample problems on a range of texture databases [62]. In this paper we propose, as many have done before [63], to use what has proved effective for image texture analysis for image semantic analysis. Below, we review textons in general and then give some details of our particular system of textons.

Textons, when the term was originally coined, were intended to correspond to qualitatively distinct image structures that were detected by pre-attentive vision [64]. Typical lists of likely textons had 5–10 candidates including edges, line segments, line endings, and junctions. Since then the meaning of texton has shifted to operationally, rather than semantically, specified categories of local image structure; and typical systems have used hundreds or thousands of textons [65]. A range of operational definitions of a texton have been proposed including: patches closer to a prototype patch than to any other of a set of prototypes [66], distinctive ordinal structures within a patch [67], distinctive patterns of response to linear filters [68].

For this work we use a system of 529 textons. Each pixel of the image is classified as manifesting one of these 529 textons in its neighbourhood. The first step in the classification process is to compute oriented Basic Image Features (oBIFs) on the basis of the responses of a bank of six 2-D derivative-of-Gaussian linear filters. These linear filters are a good model of the responses of V1 simple cells [69]. Based on the responses of these filters, when centred on a pixel, the pixel is classified into one of 23 different oBIF classes: flat, light blob, dark blob, eight orientations of slope, four orientations of light line, four orientations of dark line, four orientations of saddle [70]. oBIFs, it will be observed, roughly correspond to the original idea of texton. However, partitioning local image patch space into only 23 bins does not lead to histogram encodings with the greatest semantic grip: generally systems with 100–1000 textons are found to work best. To produce a larger number of textons, based on oBIFs, we compute oBIFs at two filter scales (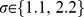) and consider the ordered pair of the fine scale oBIF and the coarser scale oBIF to specify the texton at a pixel, giving 529 = 23^2^ possible textons in our system.

To compute the similarity of two images we compare their histogram encodings using the Bhattacharyya distance [71] as in our previous work [61], [62]. The Bhattacharyya distance is a standard cosine distance, but operates on the square-rooted rather than raw histograms. Let *u* and *v* be colour or texton histograms, with their values normalized so that they have unit sum. Treat *u* and *v* as vectors. Then 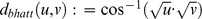. Square-rooting makes the dimensions of the representation approximately homoscedastic, which prevents well-populated bins from having excessive influence on the distance.

Recapping what was said at the beginning of the section, image similarities (computed as Bhattacharyya distances) are used to compute appearance similarities. Appearance similarities are computed as the mean similarity between each image in an appearance set to the most similar image in the other appearance set. Each appearance is modelled by a set of 50 images.

### Distributional Similarity

A precise implementation of distributional similarity is needed for a computational experiment. Choices need to be made about (i) exactly what a context is, (ii) how a distribution of contexts will be represented, (iii) how distributions of contexts will be compared, and (iv) what data source will be used to compute distributional statistics.

For data source we use the British National Corpus (BNC) [45] which is made up of written texts and transcribed speech. The text is pre-processed to remove punctuation, parentheses and unclear utterances. The words are converted to standardized word tokens, with consistent conjugation, pluralisation etc. (e.g. ‘mouse’ replaces ‘mice’). This yields 4.2×10^5^ distinct word tokens spread over 9.6×10^7^ words. The 1^st^, 10^th^, 100^th^, 1000^th^, 10,000^th^ and 100,000^th^ most common word tokens are ‘the’, ‘he’, ‘between’, ‘sorry’, ‘tenor’ and ‘uniimog’; and they occur 6.0×10^6^, 1.2×10^6^, 9.1×10^4^, 1.1×10^3^, 420 and 6 times respectively. The words we use in the experiment (*W_660_*) occur in the corpus with varying frequency. The rarest is ‘jack-in-the-box’ which occurs 12 times, the most common is ‘people’ which occurs 1.2×10^5^ times. The median frequency is 1436.

Our choices for definition for context, representation of distributions of contexts and comparison of these distributions are guided by two factors. First we hope to get good correlation between distributional and appearance similarity. Second we want the computations needed to be plausibly implemented by children’s brains during language acquisition. Based on these considerations we have choosen to use the COALS-14K method for computing distributional similarity [20].

COALS stands for ‘Correlated Occurrence Analogue to Lexical Semantics’; 14K is the dimensionality of the vector of values used to represent contextual statistics. Each vector represents the distribution of contexts for a single target word. The slots of the vector represent the tendency of each of the 14K most common words (excluding approximately 300 function words such as ‘the’ and ‘two’) to appear within the contexts of the target word. Context is defined as within four tokens before or after each occurrence of the target word.

The values stored within the vectors are not simple occurrence counts. Since the rates of appearance of different words vary over so many orders of magnitude such counts are difficult to compare meaningfully between words. Instead, the values are *based* on binary correlation coefficients that express the tendency of a word to occur in the context of a target word, taking into account the two words’ independent occurrence frequencies. These binary correlation coefficients are clipped at zero, since negative values are assumed due to noise, and passed through a decelerating non-linearity (a square root function). The final square rooting step is without theoretical justification but with clear empirical effectiveness [20].

The distributional similarity between two words is computed from comparison of their 14K dimensional vectors. In particular, we compute one minus the correlation between the values of the vectors. This gives non-negative values, with smaller numbers indicating greater distributional similarity.

To provide some anchoring for the performance of *distributional* similarity (which we denote as *DST*) we have also computed the following methods of *word* similarity which are frequently used in machine learning when concordance with possible modes of human learning is not an aim:

*PTH*: The length of the shortest path between the words along the edges of the WordNet hypernymy lattice, all edges counting equally. This measure, and also *JCS* and *VEC* which are defined next, were computed using v2.06 of the implementations described in [72].

*JCS*: A refinement of *PTH* that weights edges according to frequency statistics measured on a natural language corpus: edges are shorter the more common the hyponym [73].

*VEC*: Like *DST*, this measures distributional similarity between words [15], quantified by similarity of their second order co-occurrence statistics [74], but unlike *DST* which is based on co-occurrence within small contextual neighbourhoods in a large natural language corpus, *VEC* is based on co-occurrence within larger, expertly-constructed text samples. The samples used are expanded WordNet definitions which are the concatenation of the Wordnet definition of a word and all those words linked to it in the WordNet hypernymy and holonymy lattices [75].

*NGD*: Normalized-Google-Distance estimates the semantic similarity of pairs of words based on their co-occurrence within web pages. Computation is based on the Google Hits Counts for individual words and for their conjunction [76], [77].

*ORT*: Measures orthographic-similarity i.e. similarity of the letter sequences in two words. We implement this using a metric developed for comparison of nucleotide sequences [78]. The measure gives the score for the optimal alignment between the sequences; where the score is the number of matches versus mismatches, insertions and deletions.

### Trial Algorithm

Even with the computation of distributional and appearance similarities fixed there is still freedom in how to use the AH on each trial of the identification and naming tasks. One approach, which we call PROXY, is to focus on word pairs which are highly similar distributionally and in appearance. To continue with the example of trying to identify a *pear* despite never having seen one, the PROXY approach would roughly correspond to guessing that anything unrecognized that looked sufficiently like *apple, orange* and *banana* was a *pear*. An approach at the opposite extreme, which we call FOIL, focuses on word pairs that are highly dissimilar distributionally and in appearance. For the *pear* example this roughly corresponds to guessing that unrecognized things that look very different to *trains, whales* and *waterfalls* are *pears*. We evaluate the PROXY and FOIL approaches in supplementary results, but in the main experiment we steer a middle course with an approach which we call MIRRORING that makes use of the full range of word pairs from highly similar to highly dissimilar.

**Figure 5:**
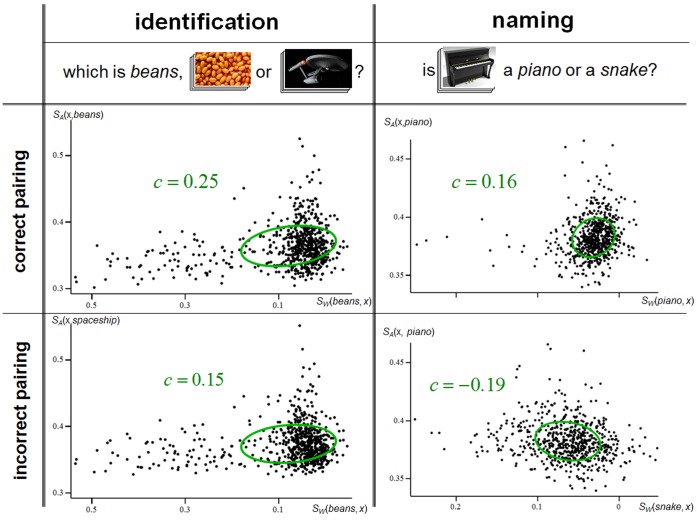
Example trials of identification and naming. The left column illustrates an example identification task, the right an example naming task. In both tasks the aim is to choose a correct pairing of a word and an appearance over an incorrect one. The plots in the upper row show data for the correct pairings, those in the lower for the incorrect. Each scatter plot relates to a different pairing of word (*W*) and appearance (*A*). For example, the top-left plot relates to 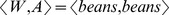, the pairing of the word *beans* with the appearance of *beans*; while the bottom-right plot relates to 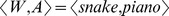, the word *snake* with the appearance of *piano*. *x* is a variable ranging over the words of known appearance. Each scatterplot has a point for each possible value of *x*. The horizontal coordinate of the points indicates 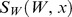, the word similarity; and the vertical coordinate indicates 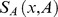 the appearance similarity. For both axes, values nearer the origin indicate greater similarity. The correlation of the points in a plot is visually indicated by the green covariance ellipses, and nearby them we give the correlation value. In both examples, the more positive correlation is in the upper row, so the correct pairing is identified.

The MIRRORING approach is based on the idea that when a word is paired with its correct appearance then the pattern of distributional similarities within the word domain should mirror the pattern of appearance similarities within the visual domain. We quantify the quality of the mirroring by the correlation coefficient between the similarity values in the two domains. When the pairing of word and appearance is correct this correlation coefficient will tend to be more positive (better mirroring) than when the pairing is incorrect. Figure 5 shows the distributional and appearance similarity data from an identification trial and a naming trial where the MIRRORING approach choose the correct answer.

PROXY approaches make use of a set of most-similar words-linked-with-appearances, and FOIL approaches use a set of least-similar. For identification tasks the set is distributionally similar to the word with unknown appearance; for naming tasks the set is appearance similar to the appearance with unknown name. For both approach we use the symbol *k* to parameterize the size of the most-similar set, and optimize *k*.

### Conditions Computed

For our main result we computed the correct rate for identification and naming tasks using word similarity based on distributional statistics, appearance similarity based on colour & texture and the MIRRORING algorithm for choosing the response to each task. We assessed the effect of the system already knowing 2, 4,…,256, 512 or 658 appearances. Each assessment was based on performance in 10^5^ trials. Separately in every trial, the appropriate number of words was randomly selected from the full set of 660 to be the already-known appearances. Also randomly selected were a further two words: call these *C* and *R* for correct and rival. For identification, the task for the system was to guess whether word *C* should be paired with the appearance of *C* or the appearance of *R*; for naming, whether the word *C* or the word *R* should be paired with the appearance of *C*.

For supplementary analysis we varied several aspects of our main computation and looked at how task performance changed. For word similarity we used other measures (*PTH*, *JCS*, *VEC*, *NGD* and *ORT*) in addition to distributional based (DST). For appearance similarity we used colour alone and textons alone in addition to both together. For task algorithm we used PROXY and FOIL in addition to MIRRORING. In total we evaluated 54 = 6×3×3 combinations.

For the variants in appearance similarity, colour alone and textons alone work as described earlier i.e. appearance similarity is based on image similarity, and image similarity is based on comparison of colour or texton histograms. For colour and texture combined, the main condition, we found that the most effective way to combine them was at the task stage rather than when computing pairwise image similarities. Specifically, in each task we computed answers using colour similarity and texton similarity separately. We then determined which of the two types of similarity gave the more unequivocal answer and used that. For example, if we were using MIRRORING and colour produced correlations of 0.3 and 0.1 for the two possible pairings, whereas textons produced correlations of 0.4 and 0.5, we would follow the colour-based scores (since 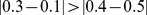) and so choose the first pairing.

For the PROXY and FOIL strategies, the parameter *k* was optimized. For small numbers of already-known appearances 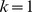 was optimal for both strategies, but optimal *k* increased with the number of appearances already-known. For PROXY it rose to 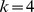 for 658 already-known; for FOIL it rose to 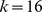.

## Results

**Figure 6:**
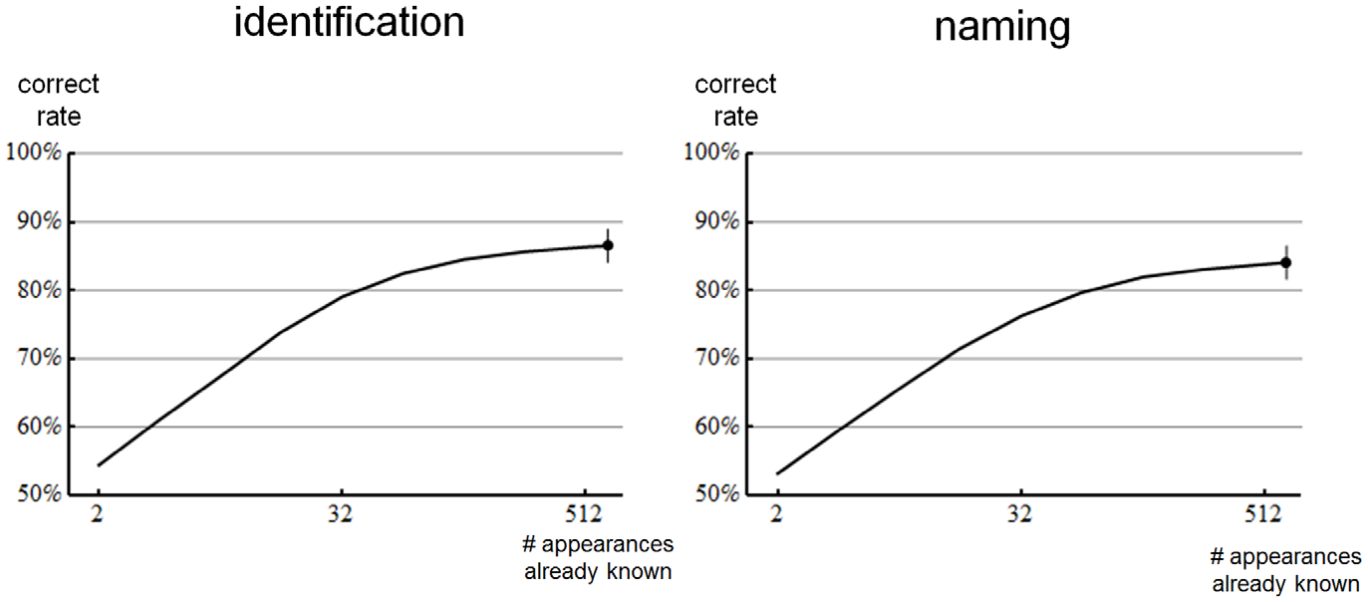
Identification and naming results. Plots show correct rates versus number of already-known appearances for the identification and naming tasks illustrated in figure 3. The baseline chance performance rate for these tasks is 50%. The response to each trial was choosen using the MIRRORING strategy illustrated in figure 5, with the word similarity (*S_W_*) being implemented using Distributional Similarity (*DST*) and the appearance similarity (*S_A_*) being implemented using Colour and Textons. At the highest point of the curves an error bar indicates the 95% confidence interval for the maximum performance obtained. Other confidence intervals are not shown but are no larger in magnitude.

Even with only two appearances already known to it, the computer system we have described, using distributional similarities (*DST*), colour & texture and the MIRRORING algorithm achieves 54% correct at the identification task and 53% at the naming task. Both scores are marginally above the baseline chance levels of 50%. As figure 6 shows, as the number of already-known appearances increases, so do the performance scores; reaching 87% and 84% for identification and naming respectively for the maximum of 658 already-known.

We have computed confidence intervals for the performance rates plotted in figure 6 using bootstrap resampling [79] of words used, images in each appearance set, and trials. In all cases the 95% confidence intervals are no greater than ±2.5%. Additionally, we have confirmed that 50% is the true baseline by repeatedly randomly permuted the pairing of words and appearances, and recomputing results. After permuting, mean performance at either task was 50.0% with a standard deviation of 0.4%. These statistical tests show that the performance we have demonstrated is significantly better than chance.

Our best results are still well short of 100%, so there is a possibility that the scores seen arise from a heterogenous performance across the set of words used. For example, maybe the AH is strongly true for animal words and untrue for non-animal; or maybe it is strongly true for highly specific words such as *shepherdess* and *wheelbarrow* and untrue for others. To assess this we have computed the identification and naming rates separately for each word as it takes the role *C*, in combination with every other word taking the role *R*. The rates were computed using 658 already-known appearances. The identification and naming scores were averaged together. The uniformity or otherwise of these word-specific scores was assessed by stratifying the set of words in two ways: by a partitioning into 21 categories (e.g. PLANT, DEVICE, etc.), and by semantic depth which is low for semantically coarser words such as *animal*, and higher for more specific words such as *squirrel*. Depth was quantified using the hypernymy path distance from the root node ‘entity’ in WordNet.

**Figure 7:**
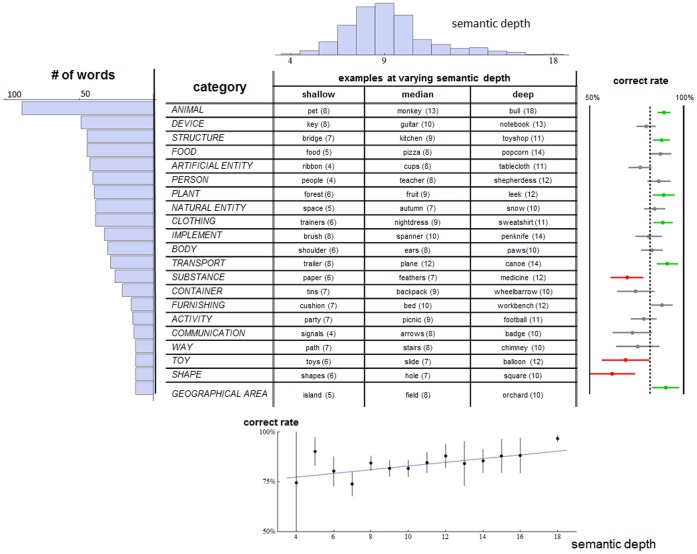
Words used in the experiment with variation of performance by category and semantic depth. The figure is organized by category varying vertically, and semantic depth varying horizontally. The histogram at top shows the distribution of depths for the full set of words in *W_660_*. The histogram at left, together with the leftmost column of the table, shows the number of words in each category. The other columns give example words (and their depths) for each category. The plots to the right and below show experimental results. At right is shown the word-specific correct rate averaged across the category. The error bars show the 95% confidence intervals of these means. The dashed line is the mean across all words. Categories with means significantly below average have red symbols, significantly above green, others grey. The plot below the table shows mean word-specific performance as a function of semantic depth. Error bars indicate 95% confidence intervals for the means. The best-fit linear function is overlaid.

The results by category are shown at the right of figure 7. The only category whose mean performance is not significantly above chance is *SHAPE*. Of the other twenty, *SUBSTANCE* and *TOY* have mean performance significantly lower than the mean for all categories; and *ANIMAL*, *STRUCTURE*, *PLANT*, *CLOTHING*, *TRANSPORT* and *GEOGRAPHICAL AREA* significantly greater. Looking at the variation across categories (s.d. 8%), together with the counts for different categories shown at the left of figure 7, we conclude that overall performance cannot be accounted for by a semantic category of words performing much better than the rest.

The results by semantic depth are shown at the bottom of figure 7. Linear regression confirms a modest upwards trend meaning that deeper (more specific) categories are slightly more easily identified and named using distributional learning; each unit increase in depth increases mean performance by 0.9% [0.3%, 1.6%]. However the effect is modest and performance is signifigantly above chance except for the very small number of words at the shallowest depth.

### Supplementary Results

**Figure 8:**
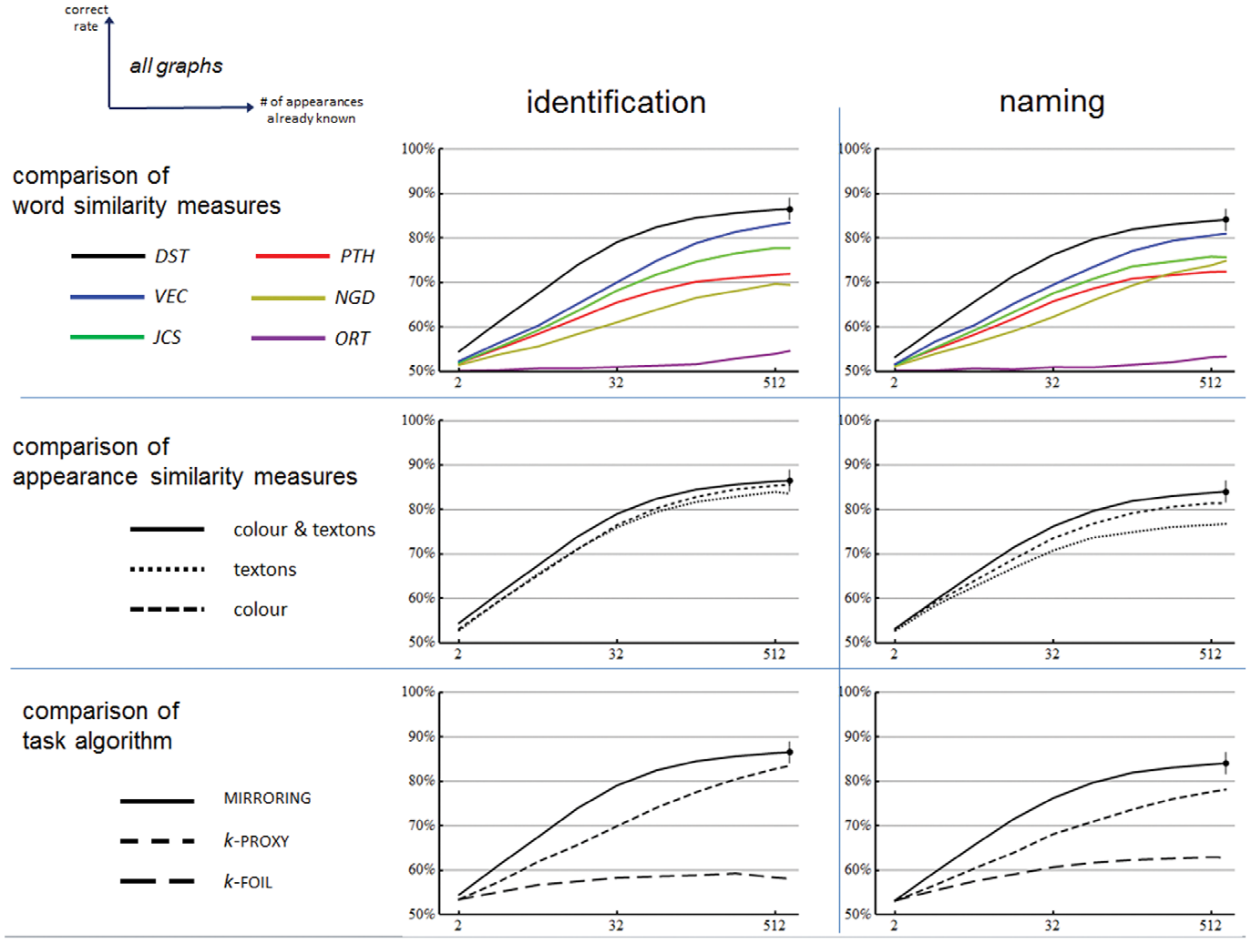
Effect of varying components of the computational system. Plots show correct rates versus number of known appearances for various combinations of word similarity, appearance similarity and trial algorithm. The unvaried components in each row are fixed at the best option. So, for example, the word similarity measure used for the middle row is *DST*. In all cases, baseline performance is 50%. Within each column, the solid black curve is the same in all plots, and the same as figure 6. At the highest point of these curves an error bar indicates the 95% confidence interval for the maximum performance obtained. Other confidence intervals are not shown but are no larger in magnitude.

Figure 8 shows the effect of varying several parts of the computational system: the measure of word similarity, the measure of appearance similarity, and the algorithm used to answer each trial on the basis of the similarities. The figure shows that for all combinations the performance at identification and naming increases with the number of appearances already known, just as it did for the main result. It also shows that the combination <*DST*, colour & texture, MIRRORING> used for the main result was the best combination. It shows that of the measures of word similarity: *DST* performs best followed in order by *VEC*, *JCS*, *NGD* and *PTH* tied, and *ORT* performs worst; but even with *ORT,* performance is significantly above chance. For appearance similarity: colour & textons together work better than either alone; and all perform significantly above chance. For task algorithm: MIRRORING is best, PROXY intermediate, and FOIL is worst; but even with FOIL, performance is significantly above chance.

Table 1 presents supplementary results showing how our results are effected by the choice of textons used for appearance similarity, and by the sources of the images used. These results are included to allow calibration against other work. To assess the influence on our results of having used Google Image to assemble image data, we instead sourced images from ImageNet, whose images have been quality controlled for label correctness. Comparison of scores C and D in Table 1 shows that we found only negligible difference. To assess the influence on our results of having used textons derived from oBIFs, we instead used textons based on the Scale Invariant Feature Transform (SIFT) [48] which are more widely known. For this we made use of the SIFT-based texton encodings available on ImageNet for images for some words. Comparison of scores A and B in Table 1 shows that oBIFs perform at least as well as SIFT. The assessments of sensitivity to image source and texture feature were made using reduced sets of categories because of data availability. Potentially performance on these reduced sets could be different than the full sets, but the similarity of scores B and C, and D and E argues against that.

**Table 1:** Effect on performance of image source and texton system used.

		Source of Images			
		ImageNet		Google Images	
Texton System		SIFT	oBIFs	SIFT	oBIFs
Word Set	*W_93_*	A: 82%	B: 83%		
	*W_420_*		C: 83%		D: 82%
	*W_660_*				E: 82%

SIFT is the Scale Invariant Feature Transform, amd oBIFs are oriented Basic Image Features – alternative methods for analyzing local image structure. To allow comparison between rows, all scores are correct rates for the identification task given 64 already-known appearances. Baseline performance is 50%; confidence intervals are not wider than ±2.5%.

## Discussion

In this discussion we relate our model to previous work, and consider its accuracy as a model of a possible mode of learning in humans.

### Relation to Previous Work

We have presented evidence in support of three findings:

1. The Appearance Hypothesis (AH): *words that occur in similar contexts tend to have referents with similar appearance*
2. By exploiting the AH a computational system can demonstrate distributionally-learnt knowledge of the appearance of words by performing better-than-chance visual identification of categories of object that it has no associationally-learnt knowledge of.
3. In the computational system, bringing all words and appearances already known to bear (with the MIRRORING algorithm) is more effective than using only the highly -similar or dissimilar.

Findings (1)–(3) have been shown to hold fairly uniformly across a diverse, large (660) set of words. Using the same numbering, the relation of these findings to previous work is as follows.

1. Correlation between lexical similarity and appearance similarity has been reported before but always with important differences from the current report: using subject-generated physical features rather than image-based visual features [80], [81]; using lexical similarity based on manually constructed semantic ontologies, or web-querying of co-occurrence, and with a limited range and number of categories [82]; using lexical similarity based on manually constructed semantic ontologies and without analysis of statistical significance [83], [84]; using lexical similarity based on manually constructed semantic ontologies [85].
2. Identification of unfamiliar objects has been previously demonstrated [82] but only with lexical similarities that are based on information sources that go beyond pure distributional, and only with a small number (10) of words, all from a narrow range (mammals).
3. The only algorithm assessed previously [82] depends on the most similar words only, so if of the PROXY type.

### Accuracy of the Model

The purpose of our computational model was to demonstrate that a distributional mode of learning appearances was a viable possibility for human language acquisition. We have presented a computational model of such a mode of learning, and so would like to conclude that the *mode* is viable. Our computational model of this mode has to use a particular algorithm to do its learning, but we do not claim that this particular algorithm is efficient or likely to be used by human learners.

Even with our limited agenda of demonstrating the viability of a particular mode of learning for humans, we have to be cautious about the conclusions that we draw since they are based on a model, not on critical observations of the real system (i.e.human learners). Conclusions based on models are only as reliable as the models are accurate. In this section we review the abstractions of our model, and consider their accuracy. We consider the tasks the system has to perform, the set of words used for testing, the computations of distributional and appearance similarity, and the data needed for those computations.

**Figure 9:**
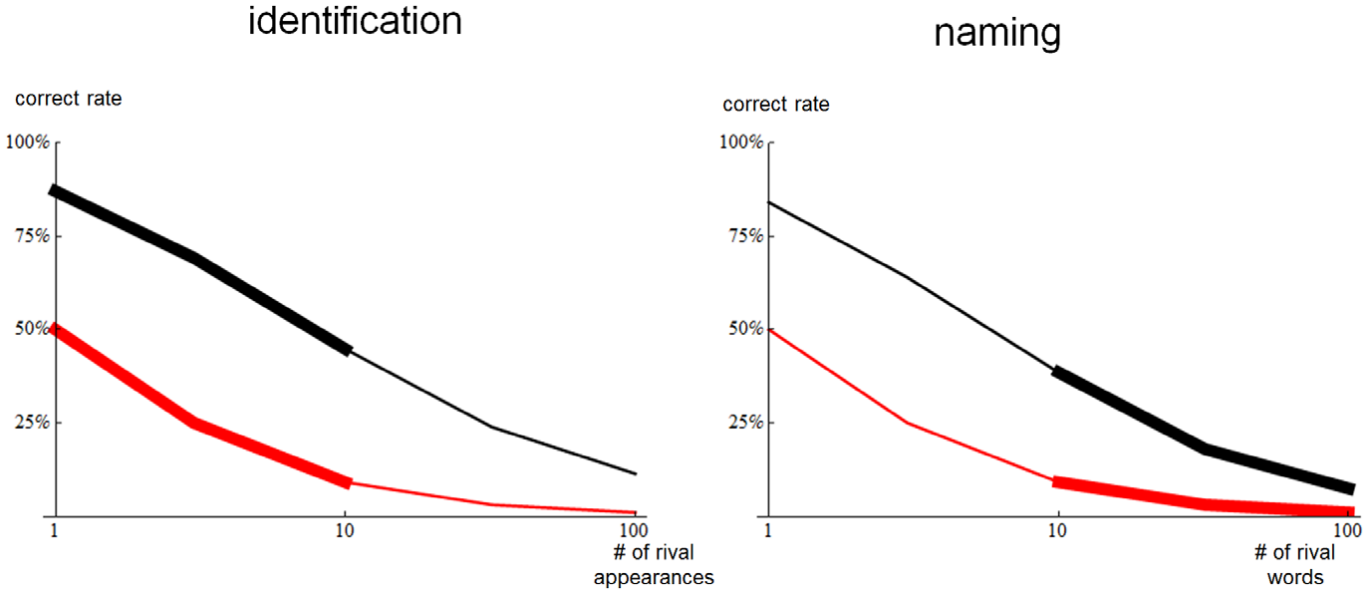
Performance as a function of the number of rival responses. In both plots, the black curve shows performance of the computational system using distributional similarities (*DST*), colour & textons for appearance, the MIRRORING algorithm and 512 already-known appearances. The red curve shows baseline chance performance. The horizontal axes show the number of incorrect rival answers that are presented alongside the correct answer. The left hand end of each horizontal axis corresponds to just one rival, which is the condition used in all other experiments (figures 6 & 7). The thicker parts of the curves indicate the range of numbers of rival that are relevant to human learning.

We first consider the realism of the identification and naming tasks our model learner is assessed on. Each trial of the identification task had only *one* rival appearance that had to be distinguished from the correct appearance. In human learning, identification scenarios (e.g. ‘pass me the trumpet’) could easily involve cluttered scenes containing *many* rival categories of object. Excluding objects that can be recognized as belonging to a known category [12], what then counts is the number of unknown categories present in the scene: the range 1–10 seems to cover most plausible scenarios. Similarly, each trial of the naming task had only one rival category name that the system had to distinguish from the correct one. In human learning, the number of rival names would be determined by the number of words with known distributional statistics but unknown appearance that the learner is carrying around in memory. We can find no data on this, but the range 10–100 seems reasonable. We have investigated how our model performs when there is more than one rival. Full results are shown in figure 9, and summary results are as follows. For the identification task, for 3 rivals, which is in the middle of the plausible range, the system gets 69% of trials correct, compared to a chance baseline of 25%. For the naming task, for 32 rivals, which is in the middle of the plausible range, the system gets 18% of trials correct, compared to a chance baseline of 3%. In both cases, distributional learning seems to offer something useful in trials which match the number of rivals of human scenarios.

Although the 660 categories we have used are a much wider set than the ten categories of mammal used in the closest previous work [82], they still account for only 2% of those known by an adult. There is evidence pointing both ways relating to what we might expect if the current experiment was scaled up to a larger set of categories. Pessimistically, Deng et al. (2010) have shown that machine vision performance does not necessarily generalize from small to larger numbers of categories [83]. Optimistically, the other 98% of words known by typical adults on reference on average more specific categories than the ones we have tested with; and the trend shown in figure 7 (bottom) was that distributional learning of appearance was slightly easier for more specific words.

Our study has ignored the effect of the order in which words are learnt. A simple computation suggests that when this is controlled, distributional learning may become much more effective, so our model as it stands will have under-estimated its viability. Specifically, we considered identification performance based on four already-known appearances. Using the standard combination <*DST*, colour & textons, MIRRORING> our learning system scores on average 61% for randomly choosen quadruples of known appearances. We then searched for the quadruple of appearances that gave the best identification performance. Searching all eight billion possible quadruples was not possible, so we looked instead for the best among 10^3^ choosen at random. We found that with the appearances of *carriages*, *lake*, *snake* and *wardrobe* already-known the system achieves an identification score of 73% – more than twice the improvement over baseline of a random quadruple of known-appearances.

The neurobiological plausibility of the computations our model learning system performs needs to be considered. For distributional similarity, we compute the co-occurrence rates of pairs of words within four words of each other, compared to their independent rates of occurrence. This is readily implementable with standard models of neural networks. The number of co-occurrence rates (660×14,000≈10^7^) is large; but since most are zero, with efficient coding and algorithms, this is easily within the capacity of available neural resources. We chose not to use the dimensionality reduction step of LSA in our procedure for computing distributional similarities to avoid any contention about whether it was neurobiologically plausible. For appearance similarity we require global histograms of quantized local colour and local image structure as measured by the output of linear filters resembling V1 simple cells [69]. Both computations are readily implementable using standard models of neural machinery.

On the issue of quantity of data, for distributional similarity we have used a 100 million word standard corpus of written and spoken English. This is undoubtedly large compared to the linguistic environment of a child. For appearance similarity, we have used 50 images per category. This does not strike us as particularly large when used to the model categories whose appearance is already known, but is large when modelling an unfamiliar category to be identified. We used so many in order to make up for the crudeness of our measures of appearance similarity. How far this number can be reduced, as more sophisticated models of appearance similarity are developed and employed, remains to be seen.

In summary, the aspects of our model that are at the greatest distance from the phenomenon of human learning concern amounts of data, particularly the size of the natural language corpus used for estimating distributional similarities. The volume of data that we are using may cause us to over-estimate the viability of a distributional mode of learning appearance. On the other hand, we have noticed one aspect – order of learning words – that we have ignored, which may have caused us to under-estimate viability.

### Conclusion

We have demonstrated that the patterns of similarity that occur within language and within appearances are sufficiently correlated to allow a distributional mode of learning the appearance part of word meaning. This mode allows some approximate knowledge of the appearance of the referents of a word to be learnt without there having been any opportunities for associationist learning of the meaning of that word. This provides a possible explanation for how it is that many viewers can identify which of the objects in figure 1 is the *adze*, and more generally for how children can learn so many appearances so quickly.

Our results only bear on the viability of such a mode of learning, not on whether children actually use such a mode. There *is* evidence that children are sensitive to some statistical aspects of language [86], and that child-directed speech of the amount experienced by a child is adequate for extracting distributional information powerful enough to infer the syntactic category of words [87], but whether children are sensitive to distributional statistics and, if they are, whether they make use of these for generalizing appearance in the way that we have described remains to be shown. Such investigations are a task for the proposed new Science of Learning grounded in Psychology, Machine Learning and Neuroscience [88].
